# Integrating forest inventory and analysis data into a LIDAR-based carbon monitoring system

**DOI:** 10.1186/1750-0680-9-3

**Published:** 2014-05-08

**Authors:** Kristofer D Johnson, Richard Birdsey, Andrew O Finley, Anu Swantaran, Ralph Dubayah, Craig Wayson, Rachel Riemann

**Affiliations:** 1 USDA Forest Service, Northern Research Station, Newtown Square, Pennsylvania, USA; 2 Departments of Forestry and Geography, Michigan State University, East Lansing, Michigan, USA; 3 Department of Geographical Sciences, University of Maryland, College Park, Maryland, USA; 4 USDA Forest Service, Northern Research Station, Troy, New York, USA

**Keywords:** Aboveground biomass, Carbon, Inter-comparison, LIDAR, Forest inventory and analysis

## Abstract

**Background:**

Forest Inventory and Analysis (FIA) data may be a valuable component of a LIDAR-based carbon monitoring system, but integration of the two observation systems is not without challenges. To explore integration methods, two wall-to-wall LIDAR-derived biomass maps were compared to FIA data at both the plot and county levels in Anne Arundel and Howard Counties in Maryland. Allometric model-related errors were also considered.

**Results:**

In areas of medium to dense biomass, the FIA data were valuable for evaluating map accuracy by comparing plot biomass to pixel values. However, at plots that were defined as “nonforest”, FIA plots had limited value because tree data was not collected even though trees may be present. When the FIA data were combined with a previous inventory that included sampling of nonforest plots, 21 to 27% of the total biomass of all trees was accounted for in nonforest conditions, resulting in a more accurate benchmark for comparing to total biomass derived from the LIDAR maps. Allometric model error was relatively small, but there was as much as 31% difference in mean biomass based on local diameter-based equations compared to regional volume-based equations, suggesting that the choice of allometric model is important.

**Conclusions:**

To be successfully integrated with LIDAR, FIA sampling would need to be enhanced to include measurements of all trees in a landscape, not just those on land defined as “forest”. Improved GPS accuracy of plot locations, intensifying data collection in small areas with few FIA plots, and other enhancements are also recommended.

## Background

Accurate, high resolution Light Detection and Ranging (LIDAR) biomass maps facilitate decision making to sequester C, for example, by identifying areas for protecting existing C stocks or planning for additional C accumulation in other areas. However, biomass maps modeled from LIDAR returns have uncertainty that should be assessed for the maps to be more useful. Forest inventories such as the U.S. Forest Service Forest Inventory and Analysis (FIA) program can be valuable for evaluating LIDAR-based and other remotely sensed biomass maps. FIA plots are systematically arranged to provide spatially unbiased estimates of forest biomass over an area, follow well-documented measurement protocols, and are quality controlled. Thus, FIA plots have been successfully used to calibrate remote sensing-based models [1-3] and provide independent estimates of biomass stocks [4,5], and biomass change [6]. The FIA plot design has also been used specifically in calibrating and validating LIDAR-derived biomass maps [7,8] and in optimizing sampling strategies to train LIDAR biomass models [9].

There are also challenges with using FIA data for biomass map evaluation since the program was not specifically designed for this purpose. First, FIA defines forest land based on both tree stocking or land use^a^, and does not usually sample areas that are considered to be “nonforest” (e.g. pastures, roads, suburban areas, parks and rights-of-way) even if trees are present [10]. In Maryland, about 25% of the aboveground carbon was estimated to be stored in “nonforest land” [11] and this discrepancy alone could account for considerable disagreement between FIA data and LIDAR mapped results. Another issue is uncertainty in the estimation of biomass from field measurements, specifically because of allometric model error and choice of allometric model to apply [12]. Finally, the FIA plot design and geolocation errors of FIA plots complicate comparisons with biomass map pixels.

**Figure 1:**
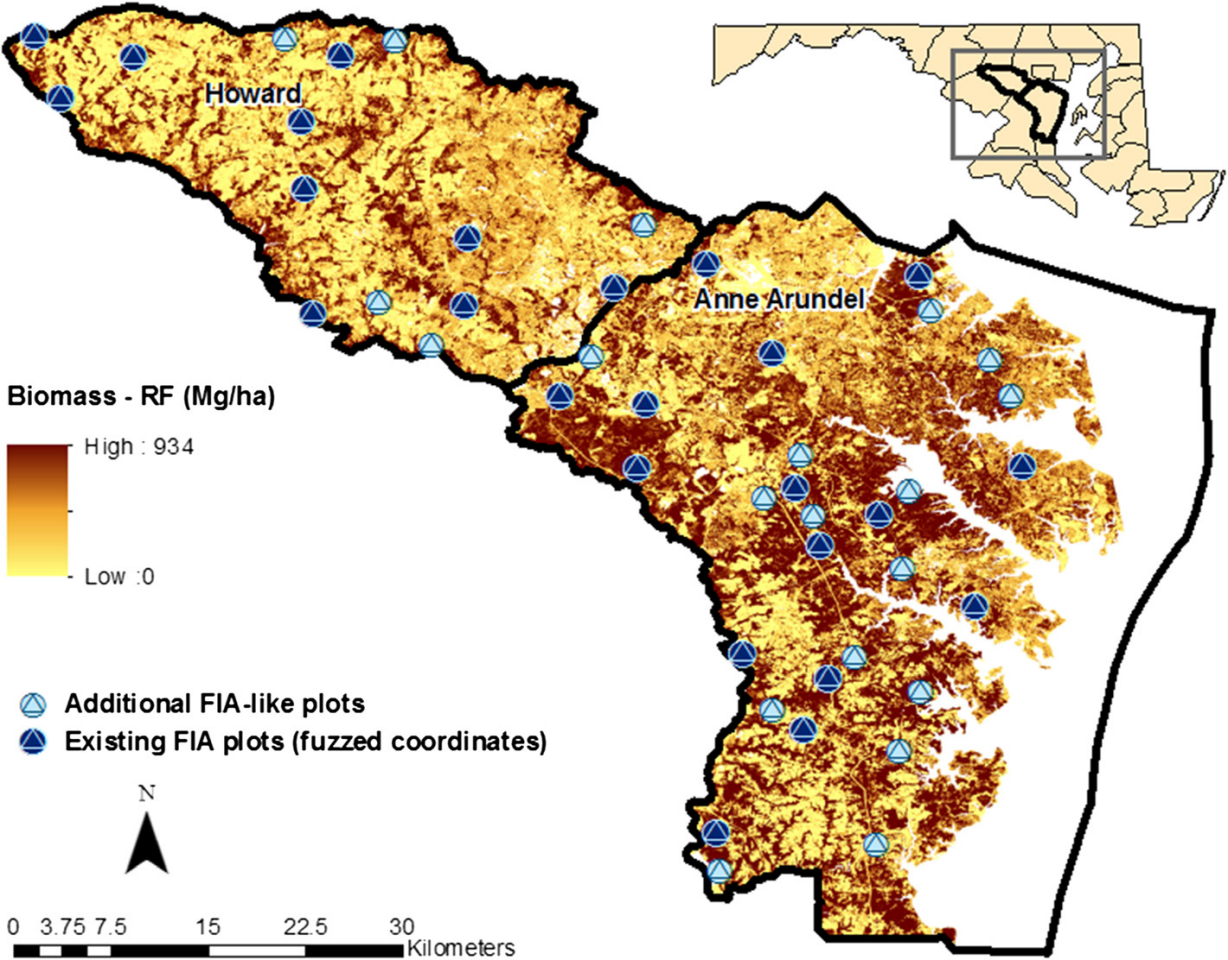
**Aboveground biomass map created with LIDAR using the Random Forest Approach (RF) for Anne Arundel and Howard Counties.** Also shown are the FIA plots and additional FIA-like plots measured in 2011 used for map evaluations.

Maryland is one of several U.S. states with statewide LIDAR available, and since 2011 the use of LIDAR-derived biomass maps has been explored for carbon monitoring purposes [13]. Unlike many large-area remote sensing biomass mapping efforts [2,3], FIA plots in Maryland were not used for the development of the LIDAR biomass prediction models in this study. Instead the FIA data in Maryland was used as an independent comparison to LIDAR-based biomass maps trained from a separate field inventory. In the current analysis we report key results about comparing FIA data with two high resolution (30-m) biomass maps, one using random forest and one using Bayesian spatial regression (see Methods) at both the plot and county scales in a case study of the Anne Arundel and Howard counties in Maryland (Figure 1). Although we include standard comparison statistics (R^2^, RMSE, etc.), the purpose was not to determine which biomass map was “better” for the two counties. Rather, we investigated issues with integrating FIA data with LIDAR-based maps by analyzing the consequences of incomplete tree data (i.e. no measurements of “nonforest” trees) and measurement error (i.e. allometric model choice and allometric model prediction error). Finally, we recommend ways that the FIA protocol could be enhanced for integration with LIDAR-based carbon monitoring, and suggest some approaches to efficiently combine the two observation systems.

## Results

### Allometric model and model choice errors

When simulated allometric model errors were propagated to each plot, they were relatively small with an average 95% confidence interval of only 5 Mg/ha (11% of the total biomass) for all the plots. One plot’s confidence interval was 93% of its total biomass, though this plot also had low biomass in absolute terms (22 Mg/ha). The mean biomass calculated from the three different allometric model choices was somewhat variable, although none of the estimates was significantly different from the others (P < 0.05). The highest estimate resulted from the Species Specific approach (mean: 208 Mg/ha, std dev: 147 Mg/ha), which was 31% higher than the CRM (mean: 159 Mg/ha, std dev: 98 Mg/ha). The Jenkins equations also produced estimates that were higher than the CRM, by 16% (mean: 184 Mg/ha, std dev: 119 Mg/ha). For the following comparisons to the LIDAR-modeled biomass map products, the Jenkins estimate was used since the Jenkins equations were applied to estimate biomass for the plot data used in the training of the LIDAR-models.

### Plot and pixel comparisons

**Figure 2:**
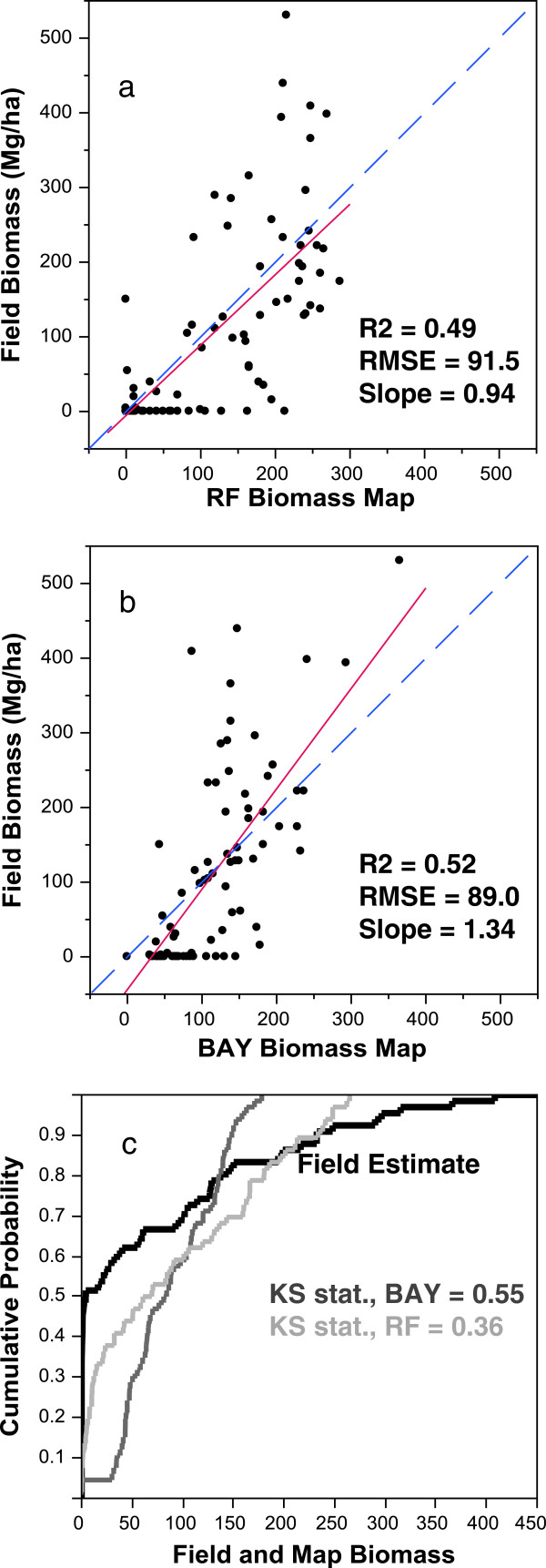
**Comparisons of biomass map pixels and field plots for the (a) RF and (b) BAY biomass maps. (c)** Comparisons of the cumulative distribution functions and respective Kolmogorov-Smirnov statistics (KS stat.) for both maps. High KS stat indicates a higher maximum difference between the distributions.

Overall, the biomass estimates from the FIA + NFI and FIA-like plots were moderately correlated with both LIDAR maps. For the RF model, the R^2^ was 0.49 with a slope of 0.94 (RMSE – 91.5 Mg/ha). For the BAY model, the R^2^ was 0.52 with a slope of 1.34 (RMSE – 89.0 Mg/ha) (Figure 2a,b). Both LIDAR maps predicted higher biomass in areas where the plot biomass measured in the field was very low or zero and yet also tended to predict lower biomass for plots with very high biomass. For example, disagreements were reflected in the comparisons of cumulative distributions (Figure 2c). Half of the field measured observations had biomass less than 1 Mg/ha, whereas half of the predicted values at the same locations were less than 68 and 81 Mg/ha for the RF and BAY models, respectively. In contrast, the mean biomass of the 5 highest biomass plots was 434 Mg/ha, compared to 228 and 240 Mg/ha for the RF and BAY models, or about half of the ground measurement. There was greater disagreement between the distributions of the BAY map (KS = 0.55) and the plot estimate than the RF map (KS = 0.34).

**Figure 3:**
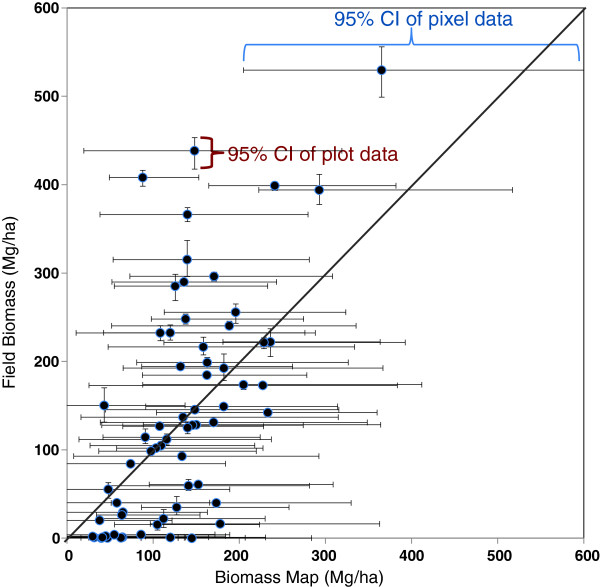
**Comparison of field measured biomass (FIA and FIA-like) and mapped biomass at the plot level, including only plots with “forest” conditions according to the FIA definition (i.e. purely “nonforest” plots were excluded).** The vertical bars are the 95% confidence interval of the mean of the field biomass values after propagating allometric and sampling errors. The horizontal bars are the mean 95% confidence levels of the LIDAR biomass map pixels for the BAY model, sampled from the posterior predictive distribution that acknowledges spatial dependence (see Methods).

When purely “nonforest” plots were removed, so that only traditionally field-measured plots were included in the regressions (n = 42), the agreement was poor. The RF map R^2^ was 0.27 with a slope of 0.91 and the BAY model R^2^ was 0.43 with a slope of 1.28. The BAY map also included pixel-level 95% confidence intervals which allowed for the comparison of 95% confidence intervals (with propagated error) of individual plot measurements (Figure 3). The mean confidence interval at the pixel level for the LIDAR model was 246 Mg/ha and 84% of the plots had a confidence interval that overlapped the confidence intervals of the corresponding pixels.

### Forest and nonforest county level estimates

County-level biomass estimated from inventory data was higher in the FIA + NFI inventory than the FIA-only inventory (Table 1a). In Anne Arundel biomass in “nonforest” conditions accounted for 27%, or 1.42 Tg, of the total. Similarly, “nonforest” biomass was 21%, or 1.35 Tg, of the total in Howard County. The biomass in Howard County was also higher than Anne Arundel for both estimates (Table 1a).

**Table 1:** County level comparisons of mean and total aboveground biomass

	**Anne Arundel**		**Howard**	
	**Mean biomass**	**Total biomass**	**Mean biomass**	**Total biomass**
a. ESTMATES FROM SAMPLED DATA	Mg/ha (95% CI)	Tg (95% CI)	Mg/ha (95% CI)	Tg (95% CI)
FIA (2006-2010)	41.4 (18.0, 65.2)	3.90 (1.66, 6.14)	74.9 (26.4, 123.5)	5.24 (1.85, 8.64)
FIA (2006-2010) + NFI (1999)	56.5 (30.5, 82.5)	5.32 (2.87, 7.76)	94.1 (41.1, 147.1)	6.59 (2.88, 10.29)
LiDAR-RF sample	86.5 (62.3, 110.7)	8.14 (5.87, 10.42)	93.5 (60.6, 126.5)	6.54 ( 4.24, 8.85)
LiDAR-BAY sample	85.2 (70.8, 99.6)	8.02 (6.67, 9.38)	89.4 (74.4, 104.3)	6.25 (5.21, 7.30)
b. ESTIMATES BY SUMMING PIXELS				
LiDAR-RF		12.89		5.65
LiDAR-BAY		11.93		5.5

The mean and total biomass was also calculated from pixel values at the plot locations for county level estimates (Table 1a). In Anne Arundel County, the LIDAR-derived value was 53 and 51% higher (about 3 Tg) than the FIA + NFI estimate for the RF and BAY maps, respectively. In contrast, for Howard County, the difference between the LIDAR-derived valued and FIA + NFI estimate was small.

When all biomass map pixels were summed and compared to total biomass estimated from field data, there were even larger discrepancies in Anne Arundel County, being well outside the 95% intervals of the FIA + NFI estimate (Table 1b). The LIDAR maps were more than twice as high in biomass, a difference of 6.95 and 5.99 Tg biomass for the RF and BAY models, respectively. In contrast, in Howard County all the LIDAR-derived county biomass estimates were well within the FIA + NFI confidence interval. Additionally, the summed pixel estimates were lower than the FIA + NFI estimate for maps in Howard, 0.94 and 1.09 Tg for the RF and BAY maps, respectively.

## Discussion

### Anne Arundel and Howard county case study

When FIA data were combined with a “nonforest” inventory, the plot data proved to be valuable for evaluation of LIDAR biomass maps in Anne Arundel and Howard Counties. Despite the low R^2^’s of both models, the comparisons still revealed that the RF model seemed to be less biased with a slope closer to 1, but tended to more severely underestimate plots with very high biomass (the reason for the lower R^2^) compared to the BAY model. Plots traditionally measured by the FIA program (i.e. no “nonforest” inventory enhancement) were also useful to evaluate LIDAR maps at the plot scale, but only for densely forested plots not confounded by plots that had both “forest and “nonforest” conditions. This comparison was aided by including the 95% confidence intervals of the LIDAR model for each pixel and the propagated allometric model and sampling errors of the plots (Figure 3).

There were significant discrepancies at the county scale, indicating that the biomass maps are predicting low biomass in areas where little or no biomass is measured. The consequence of predicting low biomass instead of none for landcovers with no trees results in comparatively larger total biomass for the counties when the pixels are summed because these areas are proportionately very large. It is unclear why the difference in Anne Arundel was so much greater than in Howard, though we note the higher proportion of agricultural landcover in Howard (30% v. 12%, determined from NLCD 2006 data). It is possible that the LIDAR biomass maps at 30-m resolution may be more successful at delineating tree v. tree-less areas in counties with higher agricultural landcover like Howard, as opposed Anne Arundel that perhaps has landcover with more fragmented tree canopies.

Using the same allometric model for both inventory and map estimates (the Jenkins equations [14]) resulted in relatively small errors compared to the choice of the LIDAR biomass model in this study. At the same time, the different allometric models led to significantly variable estimates. The CRM method has been shown to produce substantially lower biomass estimates in a number of studies due to the incorporation of tree height. For example, the 16% difference between the Jenkins and CRM methods found in this study was the same as that found on average nationally [15], but lower than the 8% difference found for Northeastern forests [16]. [12] suggested that model selection error introduced 20 to 40% to live biomass uncertainty, a range that captures the 31% difference in mean biomass between the CRM and Species Specific estimates of this study. However, these differences are less important for the purpose of map evaluation here, given that the maps used the same allometric models as the inventory for their training data.

In terms of whether the biomass maps are “accurate enough” to be recommended for carbon management purposes in these counties, it appears that one can obtain reasonable biomass values in many, but not all, areas at the plot scale (roughly 1.5 acres). Furthermore, as mentioned above, county scale estimates were only useful for Howard County, but not Anne Arundel, where more work is needed. The current evaluations have already been considered in the process of designing more effective field collection strategies and modeling approaches for developing improved biomass maps in Maryland counties. For example, newer random forest models exclude variable radius plot locations that had biomass detected by LIDAR over a 30-m area (the pixel size) but that had no trees measured in them. This can occur when trees are at the edge of a pixel, too far away to be included in the variable radius plot measurement, but still being observed by the LIDAR. When these locations were excluded the resulting model had better agreement with the FIA data because there were fewer instances where biomass was predicted in the FIA plot but there was no biomass measured (R^2^ = 0.59, RMSE = 82.4 Mg/ha, slope = 1.1; compare with Figure 2a). Another issue contributing to the poor agreement was probably our combination of a single plot design of the NFI and the regular FIA plot design, resulting in inconsistent plot-pixel comparisons throughout the sample. As “nonforest” biomass is important to consider in Maryland and elsewhere, plot designs and overall strategies for addressing the “nonforest” biomass gap, are discussed below.

## Conclusions

### Enhancing the FIA protocol by sampling trees on nonforest land

It is critical that field biomass data be both accurate and complete for evaluating biomass maps in order to improve the maps. Despite the uncertainty estimates and inconsistencies revealed by this case study, there are good reasons for integrating FIA data with LIDAR biomass maps in an aboveground carbon monitoring system. A consistent analysis would require an all-tree inventory enhancement to the current FIA protocol. This enhancement greatly facilitates comparisons at both the plot and county scales, especially in fragmented canopy landscapes that are common throughout the eastern United States. If both the maps and FIA are composed of “wall-to-wall” biomass estimates, then there is no need to distinguish “forest” from “nonforest” areas for estimating total land biomass. The main barrier to enhancing FIA data collection to include “nonforest” trees is the additional cost. An all-tree inventory would require field crews to sample trees in “nonforest” areas that are currently monitored mostly with aerial imagery. However, the cost may be lower than expected because the pre-field work imagery analysis that is already performed by FIA could screen out many plots that have essentially no chance of having tree biomass (e.g. plots located in agricultural fields). Furthermore, FIA crews already visit many “mixed condition” plots that have “nonforest” trees and so the extra time spent could be minimal, especially if only a subset of typical tree measurements are needed. The FIA program would also need to consider availability of capacity to accommodate this demand for more detailed information, but we note that cost-sharing agreements with other entities to this end have already occurred [17,18]. When a current all-tree inventory is cost prohibitive, another approach is to use previous all-tree inventories, recognizing limitations as was done in this study. For example, urban tree inventories are already available in many areas [19].

When designing an all-tree inventory to integrate into the FIA protocol, there are several alternatives to consider, each with their own set of limitations. One option is to measure the trees in all the “nonforest” conditions within the actual FIA subplots, without modifying the plot design [18]. The advantage to this approach is that newly collected data from purely nonforest plots can be easily combined with existing FIA plot data. Nonetheless, a major disadvantage to this approach is that in residential areas the four subplots will commonly cover multiple properties with different owners. Obtaining permission to visit all the subplots would therefore be more difficult and increase the chances of denied access and potentially bias the study. An alternative plot design like the one used to collect the NFI dataset of this study [20] reduces this impact on field time by sampling one larger plot instead of four. However, this design makes the total area sampled smaller and less compatible with existing FIA measurements and for relating to map pixels. A compromise between the two options is one that FIA is currently implementing in urban forest inventories, where every tree is measured in a single circular plot, located at the center of current FIA plots, and has the same area as four FIA subplots (670 m^2^) (James Westfall, personal communication). An advantage to the large continuous area is that it is much more useful for comparing to map pixels, though the design does not strictly complement the original FIA design.

### Additional enhancements and modifications

Geolocation error was not evaluated in this study but also contributes to confounding plot and pixel comparisons, especially near forest and agricultural field interfaces. For example, GPS error with the current units used by FIA is between 1 and 13 meters in heavy canopy in northeastern forests (Richard McCullough, personal communication). Thus, another enhancement to the FIA protocol would be to obtain more accurate coordinates. Though survey-grade GPS units would be ideal, even sub-meter accuracy obtained from relatively inexpensive units would be a great improvement.

In some situations it may be useful to intensify the sample size to obtain more information in areas where biomass is highest or lowest relative to the average. From our experience, it is more useful to locate the additional plots in a manner similar to the FIA design, so that the additional data are complementary for county level estimates [17]. Instead, we somewhat opportunistically located supplemental FIA-like plots in pixels indicated as forest by NLCD maps, though its stratification is not fully compatible with FIA definitions of “forest” and “nonforest”. The unintended result was that the additional FIA-like plots were located in homogeneous areas that were higher in biomass than the average FIA sample. Thus, to obtain the most information from plot intensification, a systematic design throughout the area of interest should be maintained.

Another common issue is the disparity of collection years of the different types of data. Though the error resulting from the difference in years is probably small compared to, for example, the LIDAR-biomass model error, efforts should be made to harmonize the date of LIDAR collection and the date of field data collection. Practically speaking, in the current study this would have been difficult since we were using data available to us at the time, but this should be considered in planning FIA-LIDAR data integration.

For carbon monitoring purposes, it is important to consider the discrepancies in biomass estimates from different allometric model choices [21]. The impact of allometric model choice depends on the objective for making the biomass estimate. If the estimate is used to quantify absolute biomass stocks for comparison to other counties and states, then the same allometric approach should be used in all cases. When biomass maps are used as tools for estimating biomass change in a single county, the negative consequence of choosing allometric models that are different than neighboring areas is less serious, though model selection will still have an impact. There is also unknown error when applying allometric equations developed for forestland trees, to trees located in yards and parking lots that may have different growth forms [22]. Thus, it is difficult to recommend one approach, but it is important to recognize that different allometric models can produce significantly different results, and therefore it would be useful to report estimates from more than one method or validate the selection of an allometric model with some additional field measurements of tree biomass.

Another way to improve the comparability of FIA and LIDAR estimations is to design mapping approaches that are more consistent with the ground data. For example, being careful to mimic the distribution of field measured biomass at point locations will result in a greater chance that the total biomass predicted by maps will have better agreement. Furthermore, since FIA has committed to providing biomass estimates using the CRM allometric approach, training data for making LIDAR relationships should also use this method. Additionally, providing meaningful pixel level confidence intervals (e.g. the BAY model of this study), are useful for analyzing agreement. Finally, when an all tree forest inventory is not practical, a serviceable but less ideal alternative is to exclude residential areas from LIDAR biomass maps so that they are more comparable with FIA measurements.

Finally, to achieve a robust and spatially explicit carbon monitoring system, it is most ideal for comparison purposes to have independently sampled model training and model evaluation field datasets, as was done in this study. Nevertheless, we think it is worthwhile to examine other approaches that could represent a fully integrated biomass inventory system, including assessing the uncertainties and costs. For example, it could be significantly less costly to collect all the field data needed for training and verification of biomass maps at the same time, rather than supporting two independent field efforts.

## Methods

### Study area and datasets

The study area includes the Anne Arundel and Howard counties composed mostly of oak-hickory forest [23]. The counties are almost cleanly divided by two different physiographic regions. Anne Arundel belongs to the Coastal Plain Province principally containing sandy soils at low elevation (100 ft). In contrast, Howard belongs to the Piedmont Province containing loamy and clayey soils at somewhat higher elevations (100–500 ft).

**Figure 4:**
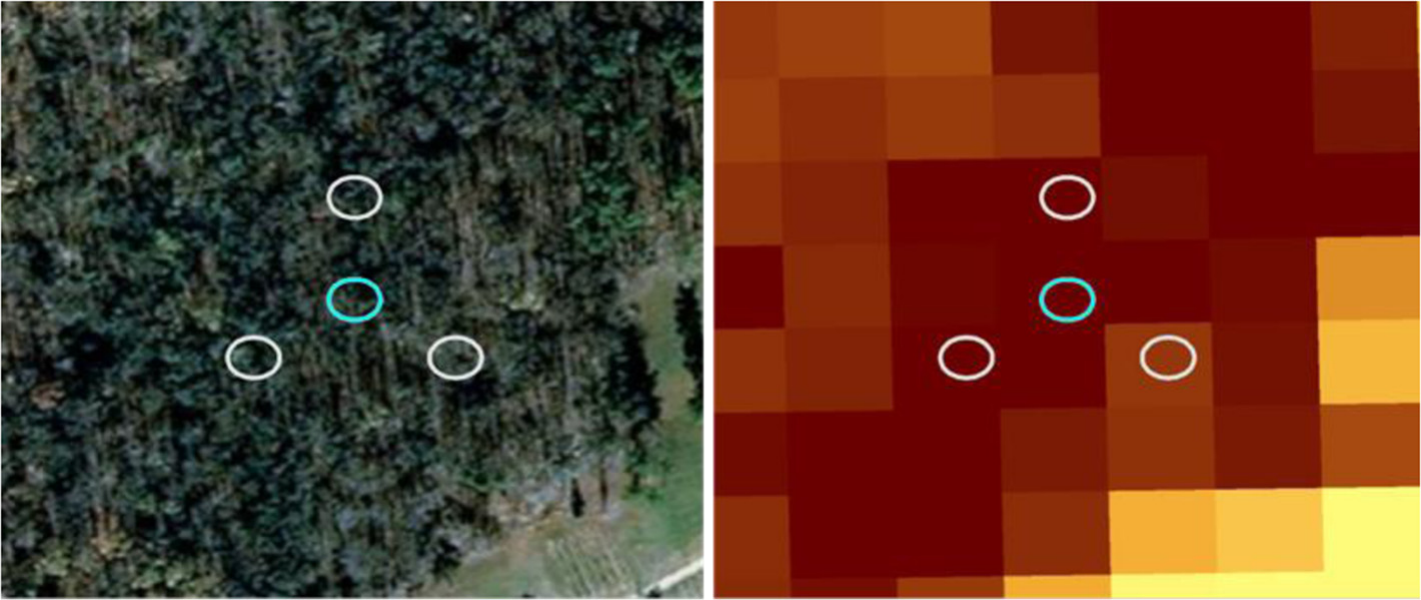
An example of the size of FIA subplots overlaid onto imagery and a biomass map of 30-m pixel resolution.

There were three field inventory datasets used to evaluate LIDAR biomass maps. Two of the inventories followed the conventional Forest Inventory and Analysis (“FIA”) plot design, that is four clustered subplots, each 168 m^2^, and spaced 7-m apart [10] (Figure 4). FIA tree level data for 64 plots within the Anne Arundel and Howard counties were downloaded from the FIA DataMart website for the 2006 to 2010 cycle period. There were a total of 72 forest plot locations, but 8 of these plots were not visited due to denied access. Of the 64 visited plots, only 9 were recorded to have purely “forest” conditions; that is, some proportion of the sample-plot area was determined to be “nonforest”. Therefore, to augment the dataset for plot-level comparisons in forested areas, an additional 20 forest plots of the same dimensions were measured in the two counties in 2011 (“FIA-like”) (Figure 1). The FIA-like plot locations were placed within forest landcover indicated by National Land Cover Database 2006 (NLCD; [24]). Finally, we took advantage of a previously collected dataset - a Nonforest Inventory (“NFI”) collected by [20] in Maryland in 1999 at FIA plots. An important nuance of the NFI dataset is that only the center subplot was measured, sampling a larger subplot area, but overall the sampled area per plot changed from 670 m^2^ to 400 m^2^. Due to the disparity in inventory years between the NFI and FIA inventories, the locations of each plot were checked with imagery one by one for evidence of clearing or forest ingrowth, but none was found. Despite the difference in inventory years, and recognizing the potential errors of combining different plot designs, for some analyses we used the NFI dataset to fill the “nonforest” biomass data gap when trees were present but not measured in the regular FIA data collection (“FIA + NFI”).

### LIDAR-derived biomass maps

Leaf-off LIDAR data collected by the Maryland Department of Natural Resources (DNR) over Anne Arundel and Howard Counties in 2004 were used to derive biomass maps for this study. LIDAR first and last returns were interpolated and differenced to obtain a normalized difference surface model (nDSM) with a resolution of 2 m. Next, a high resolution tree cover map was created by segmenting the LIDAR data and NAIP imagery [25,26] and further used to mask out everything but tree crowns on the nDSM. The resulting canopy height model (CHM) was used to calculate height percentiles, density metrics, canopy cover and other LIDAR metrics describing the vertical and spatial distribution of vegetation structure within 30 m pixels [13].

Field biomass data for developing LIDAR biomass models were collected in 300 new variable radius plots in the two counties, independently of the FIA program. Variable radius sampling is typically used to estimate basal area of a forested tract by sampling trees with probability proportional to tree basal area and is known to be a quick and accurate method for estimating stand basal area and volume [27].We collected tree measurements over variable radius plots using a model-based stratified sampling approach based on the NLCD land cover class and LIDAR height class. Field based allometric estimates of biomass, calculated using equations from [14], were then related to LIDAR variables to predict biomass using Bayesian model averaging and Random Forests regression (Figure 1).

### Random forest model

Random Forests, (RF) [28,29] is a machine learning algorithm in which a large number of regression trees are fit to a dataset (~500). Bootstrap samples are used from the data to construct each tree and at each node, a random subset of predictors are tested. Response values from all trees are averaged to provide accurate predictions and “out-of-bag” error estimates are calculated using 37% of the data in each regression tree, thus avoiding over fitting and reducing the need for cross validation. Predictions from RF regression can be used to model linear/non-linear relationships using a large number of predictor variables. The RF model of this study, using the 300 variable radius plots, had an R^2^ of 0.67 and RMSE of 73.5 Mg/ha and, similar to findings in other studies for mixed forests [7,30].

### Bayesian spatial regression model

Given ground data locations and coinciding LIDAR height metrics, we used a Bayesian spatial regression model (BAY) to make pixel-level biomass predictions. Exploratory variogram analysis showed that a non-spatial LIDAR height metric regression model did not adequately explain the spatial dependence in biomass observations, i.e., there was spatial autocorrelation among the model residuals. The presence of spatial dependence among residuals violates model assumptions which can lead to incorrect parameter and prediction inference [31]. The spatial regression model includes spatial random effects that estimate, and accommodate, this residual structure. Here, the random effects arise from a spatial Gaussian process with a covariance matrix constructed using an exponential spatial correlation function. In addition to the slope coefficients associated with the LIDAR metrics and an intercept, this model estimates a spatial correlation function decay and variance parameter, as well as the non-spatial residual variance parameter. The analysis was conducted in the spBayes R package using the spLM function [32]. This modeling framework uses a Markov chain Monte Carlo approach to generate samples from parameters' posterior distributions. Given these posterior samples, composition sampling is used to sample from the posterior predictive distribution of biomass at unobserved locations (pixels) [33]. From these pixel-level posterior distributions any error statistic can be created by simply summarizing the sets of posterior samples. In the current study, the 95% confidence levels were used to map pixel-level uncertainty in Anne Arundel and Howard Counties. Fitted values for the BAY model yield RMSE of 34.67 and an R^2^ of 0.91. Note that these values are not strictly comparable to those of the RF model because they reflect the highly flexible Gaussian process used to specify the BAY model's random effects for accurate interpolation of the observed data.

### Analysis of measurement error

**Figure 5:**
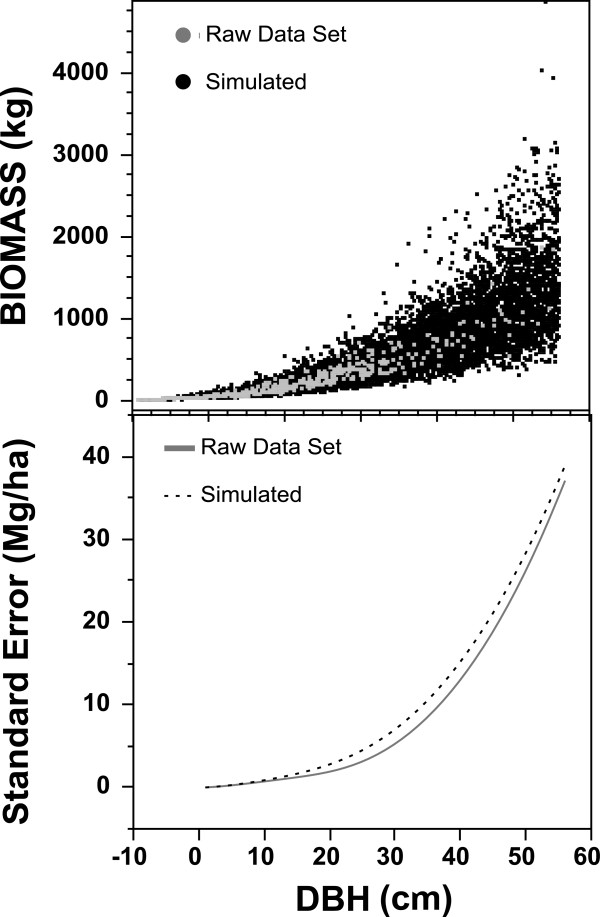
**The comparison of raw destructive harvest data from the ENFOR dataset and simulated biomass results (top panel) and the associated standard error function (bottom panel).** This example is for the “mixed hardwood” species group from [10].

To investigate allometric errors, functions for standard errors were derived by simulating a population of 10,000 data points around the regression lines of each species group published by [14]. For each species group, populations were created until the R^2^ from the regression line from the simulated points matched the R^2^ from the original equation. Next, points equaling the number of observations used in the original equations were randomly drawn from a Weibull distribution and a new standard error function was fit to the subset, where at least 100 subsets and associated standard error functions were generated. Tests of this method with actual destructive harvest data from Canada’s Energy from the Forest (ENFOR) dataset [34] showed consistent results and reflected increasing uncertainty in biomass estimates of larger trees (Figure 5). The mean of all the standard error functions for each species group was then applied on a tree by tree basis using a Monte Carlo simulation technique to calculate plot level 95% confidence intervals of the plot mean (see [35] for further details). Thus, the final plot level 95% confidence interval depended on the mixture of species groups found on the plot and their diameters.

For investigating differences in mean biomass for different allometric approaches, three sets of equations relating biomass to diameter at breast height (DBH) were applied. One set of equations was derived using the Component Ratio Method (CRM), the method used by the FIA program to report biomass stocks. The CRM equations calculate bole volume as a first step and so require “bole height” (height of the stem to 4 in diameter) in addition to DBH measurements, and the relationships are region-specific [36]. In contrast, equations applied from [14] require only a DBH measurement, are generalized for 10 species groups, and are not region-specific. Finally, yet another set of local equations, not volume-based, for species found in Maryland was used (“Species Specific”) [37-39]. In the case that no specific equation was available for a species in the Species Specific approach, the general Jenkins equation was substituted.

For comparing FIA plot measurements to mapped biomass, we chose to use the biomass equations from [14] because they represented mid-level biomass values (of the three equation types we tested) and because they were also used in the separate inventory used for the LIDAR biomass models. At the plot level, biomass map values were extracted for the coordinates of each FIA subplot from which an average of the four pixel values was calculated and compared to the ground measurement (Figure 4). The cumulative distribution functions and Kolmogorov-Smirnov (KS) statistic were calculated and compared for both the FIA observations and the biomass map observations. The KS statistic a metric of the maximum distance between the field and mapped cumulative distribution functions, where higher values reflect poorer agreement [40]. At the county level, we used two comparison approaches with the FIA + NFI data. First, we calculated the mean biomass from the pixel values extrapolated from the plot locations (in units of Mg/ha), and then multiplied the mean by the area of the county (ha) to get total biomass. This approach allowed us to mimic the FIA sample design to investigate disagreement at the plot locations. In the second approach, we calculated total biomass by simply summing the map pixels and then multiplying by 0.09 to adjust for the 30-m and 1 ha difference. We did not include the “FIA-like” plots in county level comparisons in order to maintain a systematic random sample. We also note, but do not consider in this analysis, the discrepancy between the area sampled in the field (670 m^2^) and the pixel area extracted for 4 subplots (3600 m^2^) which leads to additional errors [40]. All statistical analyses were performed using JMP [41].

### Endnote

^a^Land at least 120 feet wide and 1 acre in size with at least 10 percent cover (or equivalent stocking) by live trees of any size, including land that formerly had such tree cover and that will be naturally or artificially regenerated. Tree-covered areas in agricultural production settings, such as fruit orchards, or tree-covered areas in urban settings, such as city parks, are not considered forest land.

## Competing interests

The authors declare that they have no competing interests.

## Authors’ contributions

The study was devised by KJ, RB, AS and RD. KJ and CW performed the allometric uncertainty analysis. Comparisons were performed by KJ with ground data from RR and LIDAR biomass maps from AF, AS, and RD. All authors read and approved the final manuscript.
